# Practice pattern of use of high sensitivity troponin in the outpatient settings

**DOI:** 10.1002/clc.23482

**Published:** 2020-10-22

**Authors:** Enrico G. Ferro, Ankeet S. Bhatt, Guohai Zhou, Karen Fiumara, Jason H. Wasfy, Thomas D. Sequist, David A. Morrow, Benjamin M. Scirica

**Affiliations:** ^1^ Division of Cardiovascular Medicine Brigham and Women's Hospital Boston Massachusetts USA; ^2^ Center for Clinical Investigation Brigham and Women's Hospital Boston Massachusetts USA; ^3^ Cardiology Division, Department of Medicine Massachusetts General Hospital Boston Massachusetts USA; ^4^ Division of General Medicine and Primary Care Brigham and Women's Hospital Boston Massachusetts USA; ^5^ Department of Quality and Patient Experience, Mass General Brigham Boston Massachusetts USA; ^6^ Department of Health Care Policy Harvard Medical School Boston Massachusetts USA

**Keywords:** acute coronary syndrome, biomarkers, general clinical cardiology/adult, heart failure

## Abstract

**Background:**

High‐sensitivity troponin assays (hs‐Tn) detect lower serum concentrations than prior‐generation assays and help guide acute coronary syndrome (ACS) evaluation in emergency departments. Outpatient hs‐Tn utilization is not well described.

**Hypothesis:**

Outpatient providers use hs‐TnT to triage patients with suspected ACS.

**Methods:**

We compared the volume of outpatient prior‐generation troponin tests in the pre‐hsTn implementation period (January 2015‐March 2018) with outpatient hs‐TnT volume in the post‐implementation period (April 2018‐January 2020). Triage patterns were compared between patients with hs‐TnT≥99th vs <99th percentile, using two‐sample *t* tests. In patients triaged home, adverse events were compared between patients with hs‐TnT≥99th vs <99th percentile, using log‐rank tests.

**Results:**

Across a large tertiary healthcare system, a mean of 80 prior‐generation tests/month were ordered during the pre‐hsTn implementation period compared with 12 hs‐TnT tests/month in the post‐implementation period. Prior‐generation orders rose by 1.72 tests/month during pre‐implementation, vs a decline of 2.74 hs‐TnT tests/month during post‐implementation (*P* < .001). Among 129 hs‐TnT orders, most were placed by cardiologists (54%) and primary care providers (32%). Patient symptoms at the time of troponin ordering included dyspnea (34%) and chest pain (33%), although 25% were asymptomatic. Among symptomatic patients (n = 74), those with hs‐TnT > 99th percentile were more likely to be sent to the ED (RR, 3.36; 95% CI, 1.22‐9.25; *P* = .002). Among patients sent home (n = 66), those with hs‐TnT > 99th percentile had more adverse events by 6 months (3.3% vs 22.2% RR, 6.67; 95% CI, 1.04‐42.9; *P* = .026).

**Conclusions:**

In this healthcare system, outpatient troponin utilization significantly declined since hs‐TnT implementation. Some providers use hs‐TnT to triage patients with suspected ACS to the ED; others test asymptomatic patients and some send patients home despite high hs‐TnT values.

## 1 INTRODUCTION

Compared to prior generation troponin assays, high‐sensitivity assays for troponin (hs‐Tn) can detect biochemical evidence of myocardial injury at lower concentrations, thus improving the sensitivity for patients with suspected acute coronary syndromes (ACS), and potentially accelerating diagnosis and early therapeutic decisions.^1^, ^2^ In 2017, the Food and Drug Administration approved the first hs‐Tn assay in the United States, intended for the evaluation of patients with suspected ACS in inpatient and emergency department settings. As a result, evidence‐based algorithms have been generated for using hs‐Tn in the ACS evaluation of patients who present to the emergency department (ED) or in inpatient settings.^2^, ^3^ In contrast, there are few data describing the utilization of hs‐Tn measurements in non‐ED outpatient settings,^4^, ^5^ and in patients without ACS symptoms. We describe early practice patterns in outpatient utilization of a high sensitivity assay for troponin T (hs‐TnT) in an academic tertiary care center.

## 2 METHODS

We retrospectively identified all outpatient hs‐TnT ordered since assay implementation (post‐implementation period, 4 January 2018‐31 January 2020) and all the prior fourth generation TnT ordered in the 3 years before hs‐TnT implementation (pre‐implementation period, 1 January 2015‐31 March 2018) at the Brigham Health (which includes one academic tertiary care hospital, one community hospital and 28 affiliated clinics in Massachusetts). The rate of change in outpatient troponin orders between pre‐implementation and post‐implementation periods was compared to determine any statistically significant change in trend, using a two‐sided alpha value of 0.05.

Electronic medical records were reviewed independently by two physicians to identify primary reason for ordering hs‐TnT, as well as patient and ordering provider characteristics, and patient outcomes. The prior generation Tn assay was the Roche fourth generation troponin T assay, which was used uniformly by all sites during the pre‐implementation period. The clinical cutpoint was 0.01 ng/mL, which corresponds with the 99th percentile reference limit. Hs‐TnT were categorized as: undetectable (reported as <6 ng/L), detectable below the institutional 99th% sex‐specific cutoff (≥15 ng/L in men, ≥10 ng/L in women), and detectable ≥99th% sex‐specific cutoff. Unadjusted two‐sample *t* tests were used to compare the proportion of symptomatic patients referred to the ED between patients with hs‐TnT ≥99th% and < 99th%/undetectable (reference group). In patients sent home after outpatient encounters, log‐rank tests were used to compare adverse cardiovascular event rates (defined as time to death, first ACS or hospitalization for heart failure) for patients with hs‐TnT ≥99th% and < 99th% (reference group). Analyses were performed using the STATA software (College Station, TX). This study was approved by the institutional review board of Mass General Brigham.

## 3 RESULTS

**FIGURE 1:**
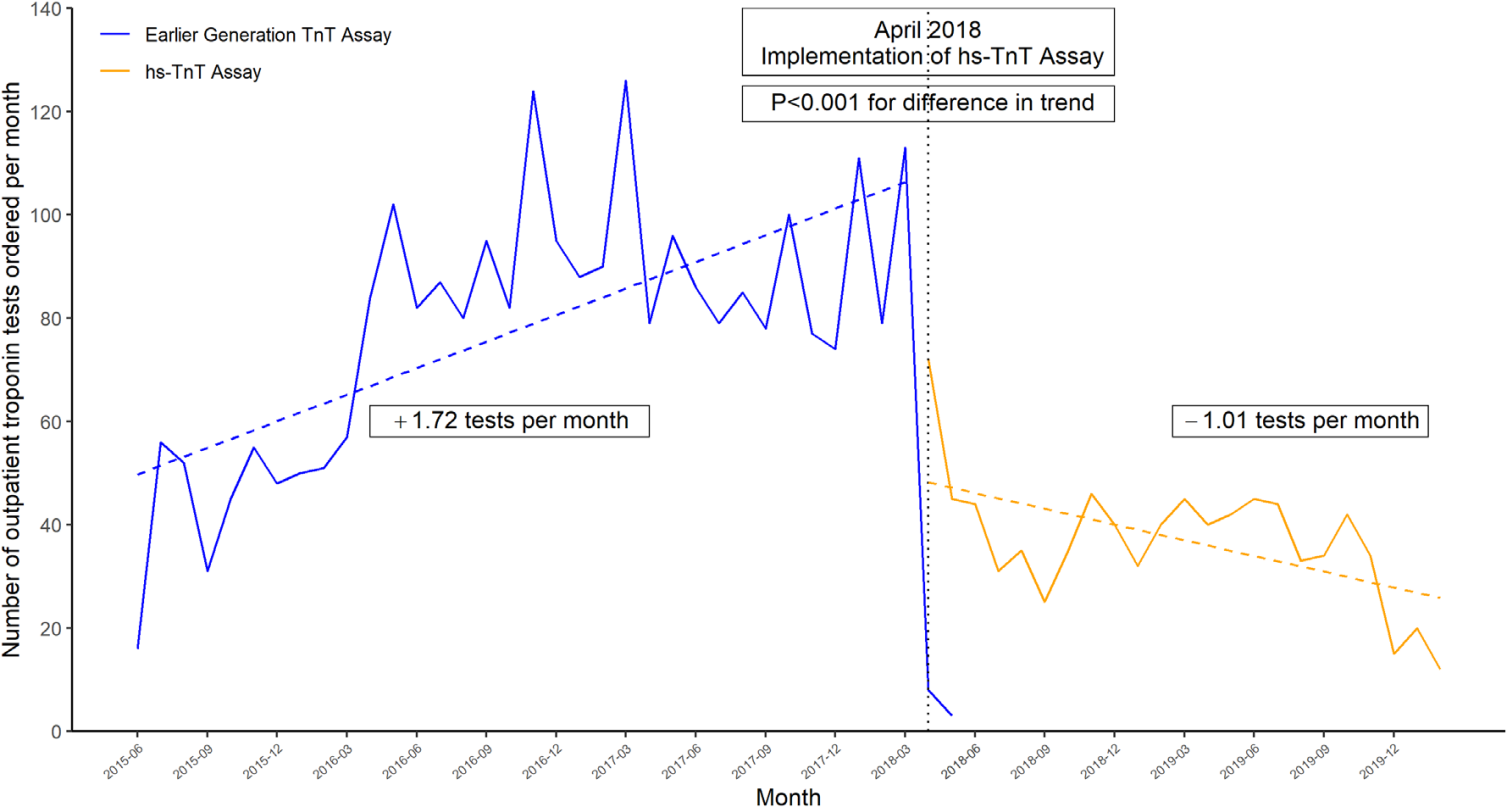
Time trend in outpatient troponin ordering. The y‐axis indicates the number of prior generation troponin assays ordered per month (blue line) and the number of hs‐TnT assays ordered per month (orange line) during the study period (x‐axis). The dotted vertical line represents the implementation of the hs‐TnT assay, and divides the study period into a pre‐implementation and post‐implementation phase. The change in slope between the two periods was compared to determine any statistically significant change in trend (as shown by the *P*‐value). hs‐TnT, high‐sensitivity troponin T

During the pre‐implementation period, outpatient providers ordered an average of 80 Tn tests/month in total across the healthcare system, with a mean increase of +1.72 tests per each month during this period (Figure 1A). During the post‐implementation period, providers ordered a total of 129 hs‐TnT tests at an average of 12 hs‐TnT tests/month across Brigham Health, with a testing rate that decreased by 1.01 tests per month during this period. The post‐implementation period had a net change of 2.74 (95% confidence interval [CI]: 1.82‐3.64, P < .001). fewer tests per month compared to the pre‐implementation period.

**FIGURE 2:**
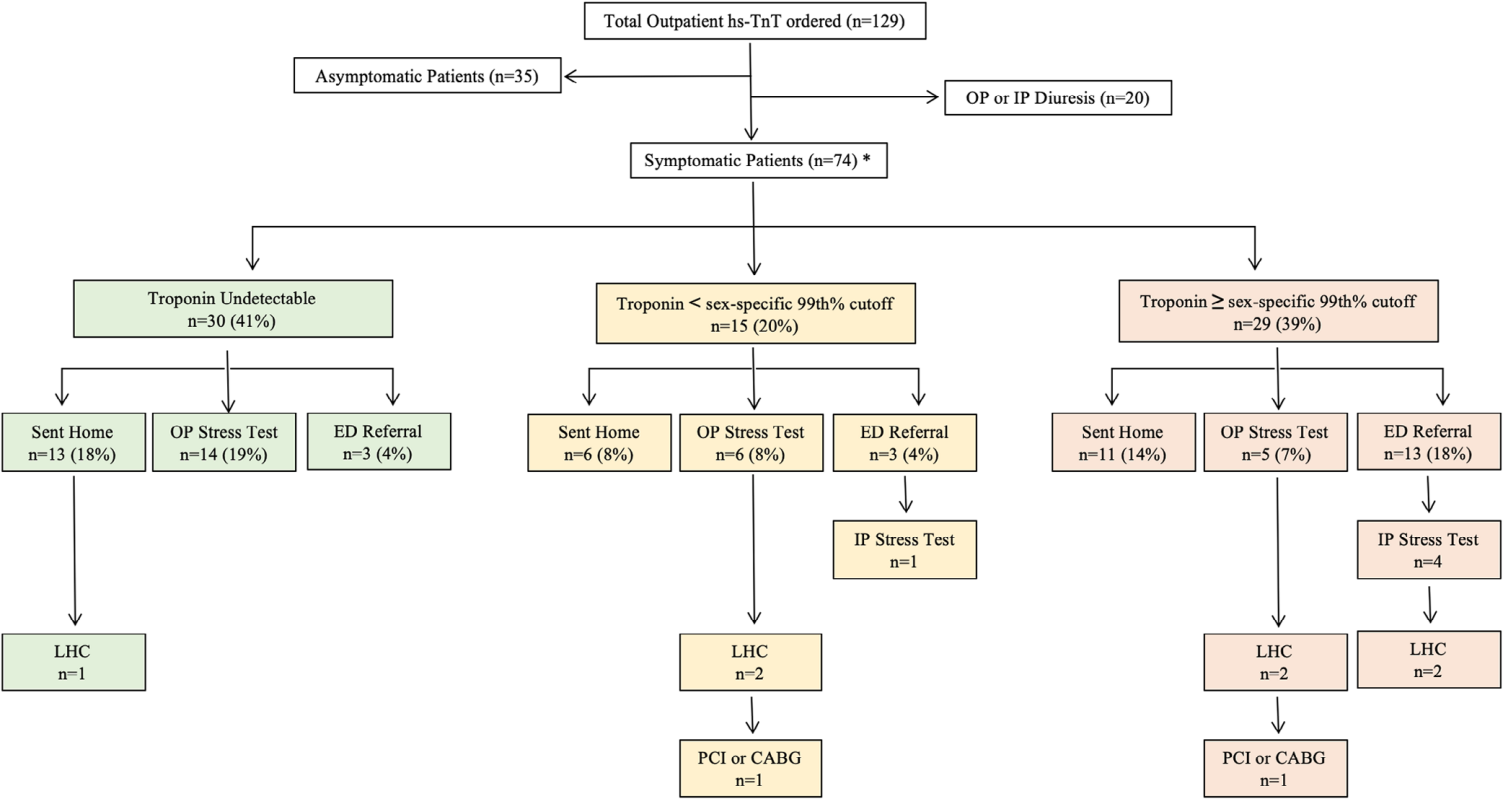
Patient triage based on outpatient hs‐TnT Level. *All percentage are calculated using “symptomatic patients (n=74)” as the denominator. CABG: coronary artery bypass grafting; ED, emergency department; hs‐TnT, high‐sensitivity troponin T; IP, inpatient; LHC, left heart catheterization; OP, outpatient; PCI, percutaneous coronary intervention

Outpatient hs‐TnT tests were most commonly ordered for dyspnea (34.1%) and chest pain (33.3%). 10.7% patients were in outpatient heart failure clinics that could provide intravenous diuresis (Table 1). Mean age of patients in which outpatient hs‐TnT was ordered was 66.2 years, 51.9% were male, 31.8% had coronary artery disease and 46.5% had history of heart failure. The mean detectable hs‐TnT value was 40.8 ng/L. A total of 31% of symptomatic and 29% of asymptomatic patients had undetectable hs‐TnT (Table 1). Ordering providers were primarily cardiologists (54.3%), followed by primary care (31.8%) and ambulatory urgent care providers (7.0%) (Table 1). Overall, 52.7% of patients were sent home, 14.7% were scheduled for outpatient ischemic evaluation, and 16.4% were transferred to the ED (Figure 2). One patient declined referral to the ED.

**TABLE 1:** Baseline patient and encounter characteristics

Patient and encounter characteristics	Total outpatient hs‐TnT orders (n = 129)
Age, years, mean (IQR)	66.2 (55.0, 80.0)
Male (%)	67 (51.9)
History of coronary artery disease (%)	41 (31.8)
History of heart failure	60 (46.5)
Heart failure with ejection fraction ≤40%	14 (10.9)
Heart failure with ejection fraction 41‐49%	8 (6.2)
Heart failure with ejection fraction ≥50%	38 (29.4)
Renal function	
Serum creatinine, mg/dL, mean (IQR)	1.3 (0.8, 1.5)
Estimated glomerular filtration rate, mL/min, mean (IQR)	65.7 (42.5, 86.3)
hs‐TnT values	
Undetectable hs‐TnT (%)	36 (27.9)
Detectable hs‐TnT (%)	93 (72.1)
Detectable hs‐TnT, ng/dL, mean (IQR)	40.8 (12.0, 57.3)
hs‐TnT orders by ordering provider (%)	
Cardiology provider	70 (54.3)
Primary care provider	41 (31.8)
Ambulatory urgent care provider	9 (7.0)
Other	9 (7.0)
Symptoms prompting hs‐TnT order (%)^a^	
Dyspnea	44 (34.1)
Chest pain	43 (33.3)
Weight gain	15 (11.6)
Dizziness or lightheadedness	10 (7.8)
Palpitations	7 (5.4)
Asymptomatic	31 (24.0)
Specialized outpatient clinic visit for intravenous diuresis	14 (10.7)
Clinical action undertaken based on hs‐TnT result	
Recommended patient to return home	68 (52.7)
Recommended outpatient ischemic evaluation	19 (14.7)
Recommended transfer to ED	21 (16.4)
Recommended direct admission	7 (5.5)
Continued intravenous diuresis in an outpatient clinic	14 (10.7)

Abbreviations: ED, emergency department; hs‐TnT, high sensitivity assay for troponin T; IQR, interquartile range.

^a^ Symptoms are not mutually exclusive, as patients may present with more than one symptom.

In unadjusted analyses of symptomatic patients (n = 74), patients with hs‐TnT ≥99th% were significantly more likely to be transferred to the ED (RR, 3.36; 95% CI, 1.22‐9.25; *P* = .002) (Table 2). Among all patients sent home after initial outpatient encounter (N = 66), those with hs‐TnT >99th% were significantly more likely to experience an adverse event in the subsequent 6 months (3.3% vs 22.2% RR, 6.67; 95% CI, 1.04‐42.9; *P* = .026) (Table 2).

Lastly, almost a quarter of patients (n = 35, 24.0%) were asymptomatic at the time of hs‐TnT ordering: the majority of them (66.7%) had elevated hs‐TnT ≥99th%, while 23.3% and 10% had undetectable and < 99th% hs‐TnT, respectively. The majority of them (83.3%) were sent home, while 10% and 6.7% were referred for outpatient ischemic evaluation and ED evaluation, respectively. Overall, 10% of asymptomatic patients experienced an adverse event in the subsequent 6 months (data not shown).

**TABLE 2:** Clinical outcomes

Clinical triage outcome for symptomatic patients, unadjusted (n = 74)				
Outcome	Hs‐TnT <99th% (n = 45)^a^	Hs‐TnT ≥99th% (n = 29)	Relative risk (95% CI)	*P*‐value
Sent to ED	6 (13.3)	13 (44.8)	3.36 (1.22‐9.25)	.002
Sent home or OP Evaluation	39 (86.7)	16 (55.2)	0.64 (0.45‐0.91)	.002
Home	19 (42.2)	11 (37.9)	0.90 (0.48‐1.69)	.714
OP Evaluation	20 (44.4)	5 (17.2)	0.39 (0.16‐0.95)	.016
Clinical outcome among patients triaged home (n = 66)				
Outcome	Hs‐TnT <99th% (N = 30)^a^	Hs‐TnT ≥99th% (N = 36)	Relative risk (95% CI)	*P*‐value
CV Event at 6 months^b^	1 (3.3)	8 (22.2)	6.67 (1.04‐42.9)	.026

Abbreviations: ED, emergency department; Hs‐TnT, high‐sensitivity troponin T assay; OP, outpatient.

^a^ The Hs‐TnT <99th % group was used as reference for statistical analysis.

^b^ CV Event, cardiovascular event, defined as time to death, first acute coronary syndrome or hospitalization for heart failure, since index outpatient encounter.

## 4 DISCUSSION

We describe the outpatient utilization pattern of troponin assays in a large academic medical center in the United States. While there was an increase in outpatient ordering of prior generation troponin assays in the 3 years prior to implementation of the high‐sensitivity assay, the launch of hs‐TnT was associated with a subsequent significant decline in outpatient troponin utilization. Almost one quarter of patients were asymptomatic at the time of hs‐TnT ordering. Higher outpatient hs‐TnT levels were associated with a significantly higher likelihood of ED referral. Among patients sent home from clinic, higher hs‐TnT levels were associated with significantly higher short‐term cardiovascular event rates.

Citing incomplete evidence, current consensus recommendations discourage the use of this assay outside of ACS evaluation in traditional ED and inpatient care settings. Several recent studies have tried to apply potential outpatient strategies to identify patients at low risk of coronary events. A recent meta‐analysis found that a single undetectable hs‐TnT, combined with a non‐ischemic electrocardiogram, has a > 99% negative predictive value for ACS for patients presenting with chest pain in the ED. This approach, however, has not been validated or formally translated into practice guidelines given the heterogeneity in the threshold for the hs‐TnT assay,^7^ and the lack of clear guidance on how to manage patients who do not have an initially detectable troponin. Another study identified a patient population (with hsTnT ≤50 ng/L and < 5 ng/L increase on repeat measurement) who had similar rates of coronary events whether they were admitted or discharged from the ED, suggesting they may be suitable for outpatient management.^8^ None of these patient populations, however, have been studied in non‐ED outpatient settings to date. The practicalities of evaluating ACS in the outpatient settings also remain undefined—for example, there are no guidelines on where and how intensively patients should be monitored while awaiting test results, or whether single or sequential hs‐TnT testing would be required to inform patient triage. Since sequential hs‐TnT testing remains the cornerstone of ACS evaluation, the appropriate workup (including first troponin test) could be initiated in the outpatient settings if time allows, but only while simultaneously coordinating (and prioritizing) immediate transport to the ED—and not using a single hs‐TnT value to inform patient triage. Our findings of declining outpatient troponin utilization since implementation of the high‐sensitivity assay seems supportive of these guidance recommendations, and may be due to clinician unfamiliarity with the assay and appropriate concerns about interpretation of its results.

At the same time, however, our finding that 25% of hs‐TnT assays was ordered for asymptomatic patients suggests that clinicians may be using hs‐TnT for alternative indications beyond ACS—such as monitoring of chronic cardiovascular conditions. Dedicated review of each indication was not immediately available in all cases, but in certain cases, troponin levels were ordered in the context of patient with underlying heart failure, amyloidosis and chemotherapy‐induced cardiomyopathy surveillance. External data has also suggested alternative uses for troponin outside of traditional indications. For example, a secondary analysis of over 16 000 outpatients with type 2 diabetes found that elevated hs‐TnT levels were significantly associated with increased risk of cardiovascular death, myocardial infarction and hospitalization for heart failure, both in patients with or without established cardiovascular disease.^9^ Similar prognostic associations have been observed in various heart failure and cardiomyopathy populations, including amyloid.^10^, ^11^, ^12^, ^13^ To date, however, it is unclear how hs‐TnT elevation can inform therapeutic considerations for these populations, and further dedicated studies are needed to inform potential uses of hs‐TnT assays for risk‐stratification and management decisions of chronic cardiovascular disease. One retrospective analysis also found that, for primary prevention among patients with hyperlipidemia, randomization to statin therapy significantly reduced levels of troponin and was associated with the lowest risk of subsequent coronary events.^14^

This study must be interpreted in the context of its design, including the fact that it presents data from a single healthcare system, which limits the generalizability of the findings. Further limitations include the reliance on chart review for ascertainment of patient symptoms and reasons for outpatient troponin ordering, as documentation was heterogenous accross encounters. Major adverse events occuring outside of the health system would not have been availible in the electronic medical record in most cases. Overall, these limitations may inform the need for larger and more representative studies to generate formal guidance on the outpatient use of hs‐TnT for the prognosis and monitoring of both ischemic and non‐ischemic heart disease.

## 5 CONCLUSIONS

Taken together, our findings support the need to develop guidance from professional societies on the use of hs‐Tn in the outpatient settings.^4^ As highly sensitive assays for troponin become more widely available in hospital systems, the lack of systemic guidance may lead to varied use in the outpatient settings. Additional data are needed to understand if a standardized, evidenced‐based approach can be developed to inform clinical interventions based on hs‐TnT results in the outpatient settings. Until definitive evidence is generated, the currently available data suggest that hs‐TnT tests should not be used for assessment of suspected ischemic symptoms in the outpatient settings.

## CONFLICT OF INTEREST

Enrico G. Ferro: Nothing to disclose. Ankeet S. Bhatt: Honorarium from Sanofi Pasteur and support by the Heart, Lung, and Blood Institute (NHLBI) T32 postdoctoral training grant T32HL007604. Guohai Zhou: Nothing to disclose. Karen Fiumara: Nothing to disclose. Jason H. Wasfy: American Heart Association Grant 18CDA34110215. Thomas Sequist: Nothing to disclose. David A. Morrow: Grants to Brigham and Women's Hospital from Abbott laboratories, Anthos Therapeutics, AstraZeneca, Daiichi‐Sankyo, Eisai, GlaxoSmithKline, Medicines Company, Merck, Novartis, Pfizer, Quark Pharmaceuticals, Regeneron, Roche Diagnostics, Siemens, Takeda, Zora Biosciences. Consulting fees from AstraZeneca, Bayer Pharma, InCarda, Merck, Novartis, Roche Diagnostics. Benjamin M. Scirica: Institutional research grants to Brigham and Women's Hospital from AstraZeneca, Eisai, Merck, Novartis, NovoNordisk, and Pfizer. Consulting fees from AbbVie, Allergan, AstraZeneca, Boehringer Ingelheim, Eisai, Elsevier Practice Update Cardiology, Esperion, Hamni, Lexicon, Medtronic, Merck, NovoNordisk, and equity in Health [at] Scale.

## Data Availability

The first and corresponding author had full access to data, which was analyzed at Brigham and Women's Hospital. We encourage investigators interested in data sharing and collaboration to contact the corresponding author.
